# Recent advances in constructed wetlands methane reduction: Mechanisms and methods

**DOI:** 10.3389/fmicb.2023.1106332

**Published:** 2023-02-03

**Authors:** Guanlong Yu, Jundan Chen, Guoliang Wang, Huifang Chen, Jiajun Huang, Yifu Li, Wenming Wang, Fengming Song, Yuanjun Ma, Qi Wang, Miaomiao Wang, Tao Ling, Zhilai Shu, Julong Sun, Zhi Yu

**Affiliations:** ^1^School of Hydraulic and Environmental Engineering, Changsha University of Science & Technology, Changsha, China; ^2^Key Laboratory of Dongting Lake Aquatic Eco-Environmental Control and Restoration of Hunan Province, Changsha University of Science and Technology, Changsha, China; ^3^Technology Center, Hunan Pilot Yanghu Reclaimed Water Co., Ltd., Changsha, China; ^4^Technology Department, Hunan Rongantai Ecological Technology Co., Ltd., Changsha, China; ^5^Technology and Information Department, CCCC-TDC Environmental Engineering Co., Ltd., Tianjin, China; ^6^Engineering Department, China Railway Wuju Group the First Engineering Co., Ltd., Changsha, China

**Keywords:** constructed wetland, methane reduction, greenhouse gas, methanogen, methanotrophy main topic

## Abstract

Constructed wetlands (CWs) are artificial systems that use natural processes to treat wastewater containing organic pollutants. This approach has been widely applied in both developing and developed countries worldwide, providing a cost-effective method for industrial wastewater treatment and the improvement of environmental water quality. However, due to the large organic carbon inputs, CWs is produced in varying amounts of CH_4_ and have the potential to become an important contributor to global climate change. Subsequently, research on the mitigation of CH_4_ emissions by CWs is key to achieving sustainable, low-carbon dependency wastewater treatment systems. This review evaluates the current research on CH_4_ emissions from CWs through bibliometric analysis, summarizing the reported mechanisms of CH_4_ generation, transfer and oxidation in CWs. Furthermore, the important environmental factors driving CH_4_ generation in CW systems are summarized, including: temperature, water table position, oxidation reduction potential, and the effects of CW characteristics such as wetland type, plant species composition, substrate type, CW-coupled microbial fuel cell, oxygen supply, available carbon source, and salinity. This review provides guidance and novel perspectives for sustainable and effective CW management, as well as for future studies on CH_4_ reduction in CWs.

## Introduction

With rapid economic and industrial development, global climate change has become an increasingly critical concern, driven by the excessive emission of greenhouse gases (GHGs) such as carbon dioxide (CO_2_), methane (CH_4_) and nitrous oxide (Dreyfus et al., 2022). GHG emissions are continually increasing worldwide, resulting in a large amount of research which focused on methods to control GHG emissions, and the development of low-carbon systems (Nuamah et al., 2020).

Wastewater treatment is one of the most resource-intensive industrial practices. Constructed wetlands (CWs) are a well-established low-cost, energy-saving, multifunctional approach to sustainable wastewater treatment, that have been widely used for the treatment of various polluted water bodies (Yu et al., 2020, 2021). However, due to the large scale of wastewater discharged, the widespread use of CWs would have an obvious environmental consequence in terms of GHG emissions. Studies have shown that the amount of GHGs emitted from CWs is 2-to 10-fold higher than from natural wetlands (Maltais-Landry et al., 2009). Current atmospheric CH_4_ concentrations are more than 2.5-fold higher than pre-industrial levels (Zhang Q. et al., 2020), which is concerning as the global warming potential (GWP) of CH_4_ in the carbon cycle is 34-fold stronger than that of CO_2_. Therefore, despite CH_4_ being present at much lower atmospheric concentrations than CO_2_, its growth rate is considerably larger, making it one of the most important GHGs contributing to global warming (Guerrero-Cruz et al., 2021). Average CH_4_ emissions are extremely variable by reviewing 158 studies data, ranging from 0.15 to 5,220 mg/m^2^/h, which can disrupt earth radiation levels (Mander et al., 2014) It has been reported that a 1-fold increase in atmospheric CH_4_ concentration would lead to tropospheric surface warming by 0.2–0.3 degrees, presenting a serious risk to human and environmental health (Dreyfus et al., 2022). CH_4_ emissions originate from both anthropogenic and natural sources, with wetland ecosystems being the largest natural source, generating annual global CH_4_ emissions of 177–284 Tg (Zhang et al., 2021b), 82% of which originate from CWs worldwide (Nuamah et al., 2020). CH_4_ has become an important aspect of global carbon reduction, due to its potentially considerable role in future warming. According to the latest IPCC report (Dreyfus et al., 2022), in order to achieve the global temperature rise control target of 1.5°C, CH_4_ emissions should be reduced by one-third by 2030 and nearly half by 2050. Achieving this goal is a necessary requirement for sustainable social and economic development (Zhang Q. et al., 2020). and effectively controlling CH_4_ emissions from CWs is an essential aspect of reducing global CH_4_ emissions.

The CH_4_ emission by CWs is the terminal product of various processes in the production, transport, and oxidation of organic matter under anaerobic conditions (Shao et al., 2020). Methanogens and methanotrophs are important microorganisms that mediate functional communities in CWs, and are closely connected with the metabolism of CH_4_ and carbon cycle processes (Bridgham et al., 2013). Disruption to the CH_4_ source-sink balance of CWs is a direct driver of dramatic increases in atmospheric CH_4_. In CW ecosystems, plants use assimilation to fix inorganic carbon from both the air and the water column (converting it into organic carbon), while also fixing organic carbon in the water column through substrate sequestration and uptake, resulting in wetlands serving as a carbon sink (Liu et al., 2022). When wetlands are subjected to long-term anaerobic conditions, plant debris, litter and the substrate gradually convert macromolecular organic matter into CH_4_ and CO_2_, which is released into the atmosphere through anaerobic microbial metabolic activities, resulting in wetlands also serving as a carbon source (Yan et al., 2012). CO_2_ released from CWs can be captured from the atmosphere through photosynthesis, with the CO_2_ fixed within plant biomass no longer contributing to the long-term carbon sink, and CO_2_ emissions from CWs are not considered as GHG because they are the natural fate of organic matter (Guo et al., 2020). The abundance, composition and activity of methanogens and methanotrophs are important determinants of CH_4_ emissions from CWs (Ji et al., 2021). In view of this, the growth of methanogens should be inhibited in CWs, ensuring a suitable environment is provided for the survival of methanotrophs, maintaining maximum conversion of the generated CH_4_ to CO_2_ and subsequently, reducing the contribution of CWs to the GWP. To stimulate the growth of methanotrophs, plant cover serving as a powerful carbon sink is used in horizontal subsurface flow CWs (HSSFCWs) which allow O_2_ transported by the aerenchyma of plant roots (Mander et al., 2008). It is confirmed that plant diversity can also increase carbon sequestration in the substrate and substrate-based carbon sequestration not only completely offsets GWP based on CH_4_ and nitrous oxide emissions, but also simulates the conversion of CWs from a carbon source to a carbon sink (Du et al., 2018). Carbon uptake in vegetated wetlands decreases with increasing levels of salinity, mainly due to the inhibition of plant productivity (Sheng et al., 2015). Microbial transport and transformation are the main reason for the high carbon source consumption of CW-coupled microbial fuel cell (MFC) systems, with these processes regulated by microbial competition driven by environmental aspects, providing a novel approach to controlling CH_4_ emissions from wetland systems (Liu et al., 2022). The contributions of carbon “source” and “sink” functions in CWs, as well as their relationship and interactions, are crucial to the material and energy cycles within CW ecosystems, global carbon dynamics and global climate change trends (Dreyfus et al., 2022).

If CWs are designed only to consider pollutant removal, the process may not fit with the current double-carbon philosophy, highlighting the importance of constructing effective wetland systems, that can achieve high pollutant removal performance while minimizing CH_4_ emissions. There remains a lack of research on CH_4_ emissions from CWs, making it difficult to accurately determine the mechanisms and processes of CH_4_ emission from CWs. Furthermore, the important environmental control factors have not been comprehensively established, reducing the validity of CH_4_ emission reduction measures, and limiting the effectiveness of CWs. Therefore, this review was conducted to explore the available research in this field.

## Bibliometric analysis

### Data collection sources

Data was collected using the Web of Science (WOS) Core Collection database, with this review only considering the Science Citation Index Expanded (SCI-Expanded). Based on the findings of previous research by Yu et al. (2022), the keywords used to screen the available research on CH_4_ in CWs included (“constructed wetland*” OR “treatment wetland*” OR “engineered wetland*” OR “artificial wetland*” OR “reed bed*” OR “man-made wetland*”) and (“methane sequestration*” OR “methane capture*” OR “methane emission*” OR “CH_4_ capture*” OR “CH_4_ sequestration*” OR “CH_4_ emission*” OR “methane reduc*” OR “CH_4_ reduc*”). The literature type was restricted to “article” and “review,” with publications in English from 1991 to December 2021 included (1991 was the earliest publication date available in the database). After the initial data search, 108 articles investigating CH_4_ in CWs were selected. The analysis of these publications were performed using Microsoft Excel (2019), charts were generated using Origin (2019 learning version) and the S-curve was prepared using the logistic model in Loglet Lab 4. Analysis of keyword co-occurrence were performed using VOS viewer software (version 1.6.15). After exporting the 108 publications from the WOS platform in plain text form, the author keyword co-occurrence function of VOS viewer software was used to analyze keyword co-occurrence, with the thesaurus files then were merged (such as CH_4_, methanes replaced with methane) and the minimum number of co-occurrences set to 3.

## Analysis

### Publication trend

**Figure 1:**
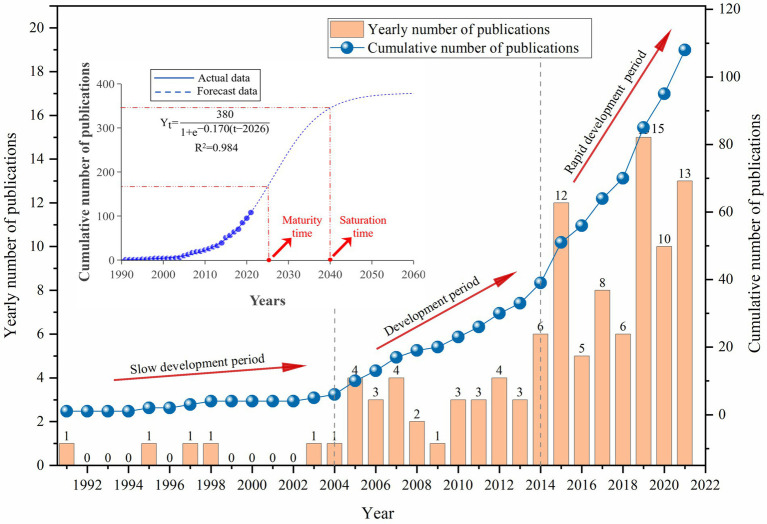
Temporal evolution of publications about CH_4_ in CWs, showing the total and cumulative trend, with the corresponding S-curve.

The publication trend for studies on CH_4_ in CWs is shown in Figure 1. In the long term, the number of publications has continually increased with fluctuations, reflecting the growing concern within the scientific community about the effect of CH_4_ emissions and the need to actively reduce CH_4_ emissions in order to maintain global ecological security (Kasak et al., 2020). Prior to 2004, the publication number was relatively low and did not increase significantly from 2005 to 2013. However, a notable increase was observed after 2014, finally reaching 108 articles in 2021. Although the number of publications on this topic remains relatively low, work in this field is gaining importance constantly, as shown by the S-curve (*R*^2^ = 0.984) in Figure 1, which indicates that research is in the growth stage and is expected to reach the inflection point in 2026 and remain stable until 2040. Therefore, publications in this field are not expected to reach saturation over the next 15 years, highlighting the high potential for innovation and development. In this sense, a comprehensive review of the current state of knowledge is essential to help stimulate and guide future development and research on CH_4_ in CWs.

### Main topic

**Figure 2:**
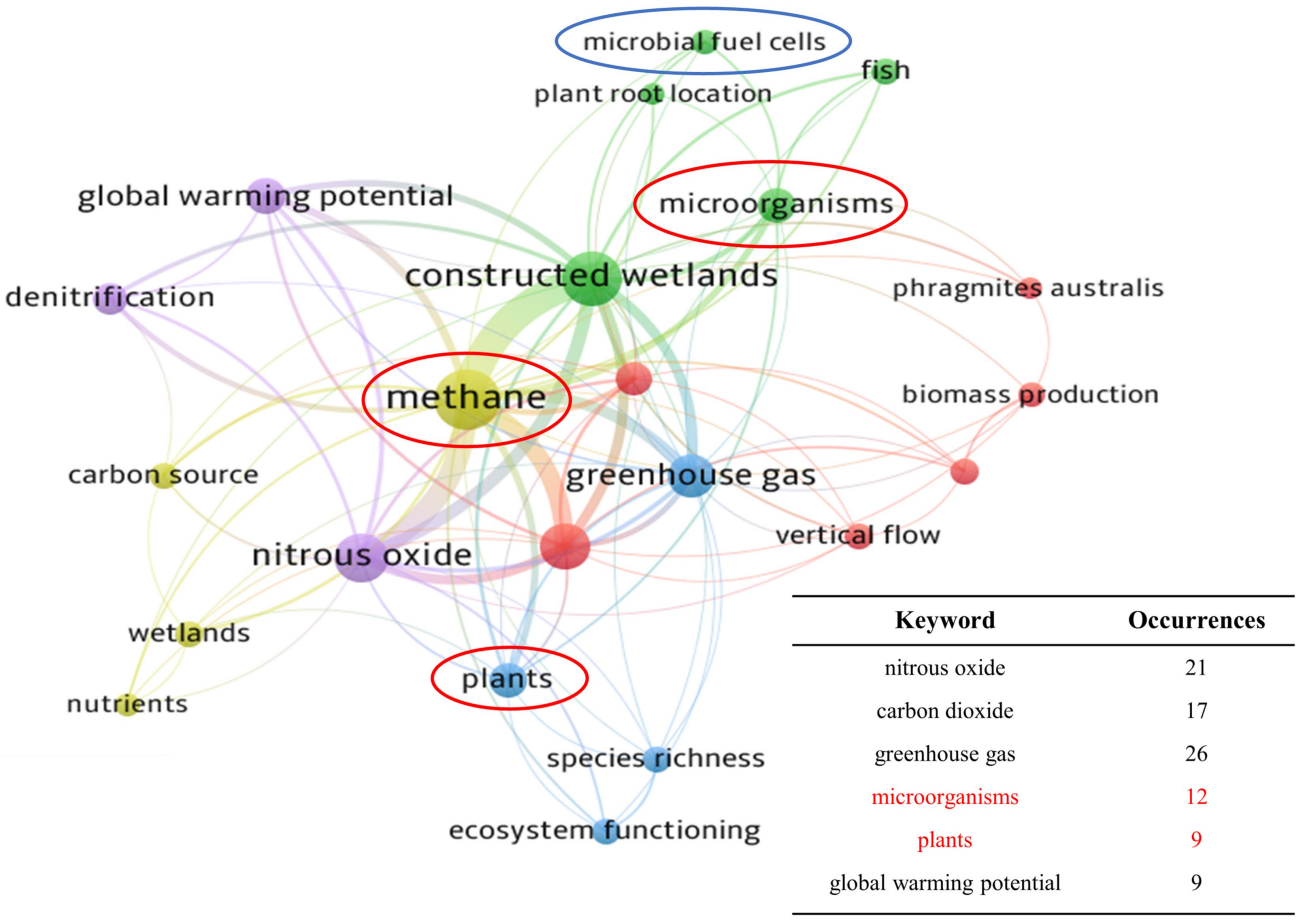
The bibliometric co-occurrence of keywords associated with CH_4_ in CWs. (Stronger degrees of connection between the keywords are indicated by closer locations and thicker lines, while a higher frequency of occurrence is shown by larger circles).

The results of keyword co-occurrence analysis are shown in Figure 2. The 6 most frequently co-occurring keywords were intercepted, showing that research on the effects of CH_4_ on GWP, plants, and microorganisms was closely linked to studies on CH_4_ emissions. Studies by Chen et al. (2020c) and Maucieri et al. (2019) attracted attention as they found that different plant species were able to exert varying effects on CH_4_ emissions. Furthermore, it has been shown that microbial diversity and abundance are a critical factor affecting CH_4_ fluxes, resulting in the need for further research and validation (Zhang et al., 2021a). MFC technology is a promising approach to the control of CH_4_ emissions from CWs. For example, Liu et al. (2022) reported that microbial competition in a CW-MFC system can convert unstable carbon sources to CO_2_ rather than CH_4_, which can considerably reduce the contribution of CWs to global CH_4_ emissions.

## Production, transport, and oxidation of CH_4_ in CWs

### Production of CH_4_ in CWs

#### Plants

Plants play a vital role in CH_4_ emissions, however due to contradictory results having been reported, the production of CH_4_ by plants was previously only considered in terms of a channel for soil-atmospheric gas exchange. However, some terrestrial plants were first demonstrated to release CH_4_ under aerobic conditions by Keppler et al. (2006), with more recent studies proving evidence that lignin, pectin, and cellulose can all serve as precursors for CH_4_ formation (Bruhn et al., 2012). The production of CH_4_ by plants has been reported as a defense strategy against environmental stress factors, such as cutting damage, increased temperatures, UV radiation, and the disturbance of cytochrome c oxidase activity (Zhang et al., 2012). Once plants trigger stress responses, ROS, such as O_2_˙^−^ and H_2_O_2_, can be overproduced within plants, further exacerbating the degradation of cellular material, leading to the production of CH_4_ (Bruhn et al., 2012; Wang et al., 2023). In addition, some of the identified CH_4_ producing terrestrial plants are used in CW applications, such as *Phragmites australis* and *Thalia dealbata*. However, CH_4_ emissions from wetland hydrophyte plants under aerobic conditions have not been investigated to date, highlighting a key gap in knowledge that requires future research.

#### Microorganisms

Anaerobic microorganisms play the major role in CH_4_ production. Under anaerobic conditions, anaerobic hydrolytic microbes, fermentative microbes, and hydrogen-producing acetogens can decompose organic matter from wastewater, substrate materials, and plant biomass (Liu et al., 2022), forming simple inorganic (e.g., CO_2_ and H_2_) and organic compounds (e.g., acetate; López et al., 2019), that are subsequently converted to CH_4_ by methanogens (specialized anaerobic archaea; Malyan et al., 2016). Anaerobic environments occur widely in the substrate layer of CWs. For example, HSSFCWs are designed to purify wastewater through anaerobic pathways (Engida et al., 2020), while anaerobic microzones have been identified in surface flow CWs (SFCWs) due to water flow layering over substrate, and vertical subsurface flow CWs (VSSFCWs) due to long-term operation causing microbial oxygen (O_2_) consumption rates to exceed the reoxygenation rate (Maucieri et al., 2017). Methanogens are divided into seven orders, belonging to *Euryarchaeota* (Table 1). Most methanogens are hydrogenotrophic, with only *Methanosarcinales* being acetoclastic (Table 1; Bridgham et al., 2013; Zhang et al., 2021c). *Methanosarcina* are fast-growing organisms that can utilize high acetate concentrations, in contrast to *Methanosaeta* (Lu Y. et al., 2015). Newly discovered methanogens have been classified as belonging to *Euryarchaeota* (such as *Methanomassiliicoccales*, *Methanofastidiosa*, and *Methanonatronarchaeia*; Dridi et al., 2012; Nobu et al., 2016; Sorokin et al., 2017), as well as *non-Euryarchaeota* (such as *Verstraetearchaeota*, *Bathyarchaeota*, and *Geoarchaeota* (Evans et al., 2015; Vanwonterghem et al., 2016; Guo et al., 2020). Based on the results of this review, three methanogenic metabolic pathways exist in CWs: H_2_/CO_2_ reduction, methyl cracking, and acetate fermentation. The hydrogenotrophic pathway is more energetically favorable to methanogenesis than the acetoclastic pathway (Zhang et al., 2018a), although the acetoclastic pathway has been reported to dominate in freshwater wetland ecosystems, accounting for more than 67% of CH_4_ emissions (Zhang et al., 2018b). Novel methanogens have also been found to utilize a fourth methanogenic pathway, involving the reduction of methyl compounds by H_2_, as originally observed in *Methanobacteriales* (Dridi et al., 2012) and *Methanomicrobiales* (Sprenger et al., 2007), then later found in *Methanomassiliicoccales* (Dridi et al., 2012), *Methanofastidiosa* (Nobu et al., 2016), *Bathyarchaeota* (Evans et al., 2015), and *Verstraetearchaeota* (Vanwonterghem et al., 2016). The specific equations are shown in Eqs 1–7.


(1) $$4H_{2}+{CO}_{2}\to {CH}_{4}+2H_{2}O$$

(2) $$4{CH}_{3}OH\to 3{CH}_{4}+{CO}_{2}+2H_{2}O$$

(3) $$2{\left({CH}_{3}\right)}_{2}S+2H_{2}O\to 3{CH}_{4}+{CO}_{2}+2H_{2}S$$

(4) $$2{\left({CH}_{3}\right)}_{2}NH+2H_{2}O\to 3{CH}_{4}+{CO}_{2}+2{NH}_{3}$$

(5) $${CH}_{3}\mathrm{COOH}\to {CH}_{4}+{CO}_{2}$$

(6) $$4{CH}_{3}\mathrm{COCOOH}+2H_{2}O\to 5{CH}_{4}+7{CO}_{2}$$

(7) $${CH}_{3}OH+H_{2}\to {CH}_{4}+H_{2}O$$

**Table 1:** Taxonomy of major methanogens in *Euryarchaeota* phylum.

Class	Order	Family	Genus	Major CH_4_ production pathway
*Methanobacteria*	*Methanobacteriales*	*Methanobacteriaceae*	*Methanobacterium*	Hyrogenotrophic,Methylotrophic
			*Methanobrevibacter*	Hyrogenotrophic,Methylotrophic
			*Methanosphaera*	Hyrogenotrophic,Methylotrophic
			*Methanothermobacter*	Hyrogenotrophic,Methylotrophic
		*Methanothermaceae*	*Methanothermus*	Hyrogenotrophic,Methylotrophic
*Methanococci*	*Methanococcales*	*Methanococcaceae*	*Methanococcus*	Hyrogenotrophic,Methylotrophic
			*Methanothermococcus*	Hyrogenotrophic,Methylotrophic
		*Methanocaldococcaceae*	*Methanocaldococcus*	Hyrogenotrophic
			*Methanotorris*	Hyrogenotrophic
*Methanomicrobia*	*Methanomicrobiales*	*Methanomicrobiaceae*	*Methanoculleus*	Hyrogenotrophic
			*Methanomicrobium*	Hyrogenotrophic
			*Methanofollis*	Hyrogenotrophic
			*Methanogenium*	Hyrogenotrophic
			*Methanolacinia*	Hyrogenotrophic
			*Methanoplanus*	Hyrogenotrophic
		*Methanospirillaceae*	*Methanospirllum*	Hyrogenotrophic
		*Methanocorpusculaceae*	*Methanocorpusculum*	Hyrogenotrophic
		*Methanoregulaceae*	*Methanolinea*	Hyrogenotrophic
			*Methanoregula*	Hyrogenotrophic
			*Methanosphaerula*	Hyrogenotrophic
		*Methanocalculaceae*	*Methanocalculus*	Hyrogenotrophic
	*Methanosarcinales*	*Methanosarcinaceae*	*Methanosarcina*	Hyrogenotrophic, Aceticlastic, Methylotrophic
			*Methanococcoides*	Aceticlastic, Methylotrophic
			*Methanohalobium*	Aceticlastic, Methylotrophic
			*Methanohalophilus*	Aceticlastic, Methylotrophic
			*Methanolobus*	Methylotrophic
			*Methanomethylovorans*	Methylotrophic
			*Methanimicrococcus*	Methylotrophic
			*Methanosalsum*	Methylotrophic
		*Methanosaetaceae*	*Methanosaeta*	Aceticlastic
		*Methermicoccaceae*	*Methermicoocus*	Methylotrophic
		*Methanotrichaceae*	*Methanothrix*	Aceticlastic
	*Methanocellales*	*Methanocellaceae*	*Methanocella*	Hyrogenotrophic
*Methanopyri*	*Methanopyrales*	*Methanopyraceae*	*Methanopyrus*	Hyrogenotrophic
*Thermoplasmata*	*Methanomassiliicoccales*	*Methanomassiliicoccaceae*	*Methanomassiliicoccus*	Methylotrophic

Malyan et al. (2016).

### Transport of CH_4_ in CWs

**Figure 3:**
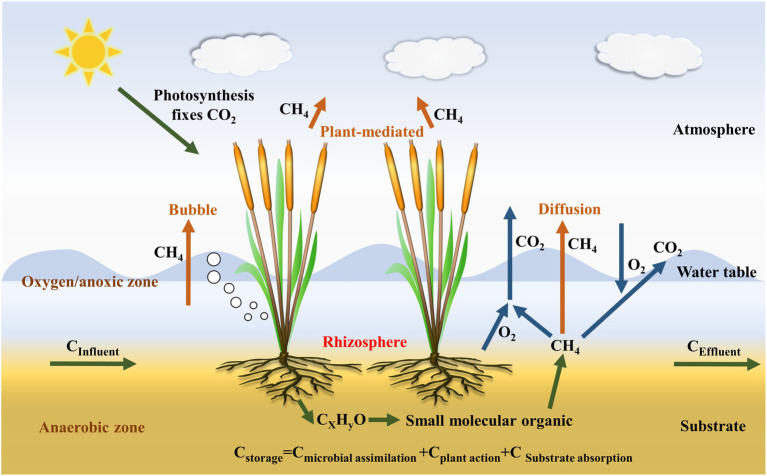
Methane production, transport, and oxidation processes in CWs.

The transport of CH_4_ occurs mainly *via* three processes: (1) direct CH_4_ transport through molecular diffusion from the water and substrate column; (2) direct CH_4_ transport through the ebullition flux process from substrate column; and (3) plant-mediated CH_4_ transport from the substrate column *via* plant aerenchym (Figure 3; Thangarajan et al., 2013; Kasak et al., 2020). Ebullition plays a major role in direct CH_4_ transport process, producing three-fold more CH_4_ fluxes than molecular diffusion by Bonetti et al. (2021), while plant-mediated CH_4_ transport is the dominant mode of CH_4_ release among three processes (Liu et al., 2017), accounting for about 70% of the total-CH_4_ emissions (Li et al., 2010). Moreover, the plant-mediated transport can also transport O_2_ to plant organ (Silvey et al., 2019). Therefore, investigations into the plant-mediated transport process are required to further our understanding of the role of plants in CW CH_4_ contributions. Plant-mediated CH_4_ transport mechanisms can be classified as molecular diffusion or convective transport processes. Molecular diffusion rates depend primarily on the CH_4_ gradient between plant roots and above-ground parts, along with the interior of the plant organ and the atmosphere, with the capacity for diffusion being susceptible to ambient temperatures (Joabsson et al., 1999). In contrast, gas movement by convective transport relies on the pressure difference between the inner-and outer-plant environment (Orr, 1992). Different plant species exhibit a diverse range of CH_4_ transport processes, with convective transport processes typically more effective than molecular diffusion processes (Xu H. et al., 2021). In a recent study by Feng et al. (2022), a novel plant-girdling method was developed, removing the epidermis and subepidermal sclerenchyma to disrupt the O_2_ transport pathway, suppressing O_2_ release and increasing CH_4_ emissions, verifying that wetland plants aerenchyma play a vital role in the transport of CH_4_.

### Oxidation of CH_4_ in CWs

During CH_4_ production and transport processes, CH_4_ oxidation is a significant factor affecting CH_4_ fluxes. CH_4_ oxidation can occur *via* aerobic or anaerobic pathways. Aerobic oxidation of CH_4_ (Table 2) mainly occurs at micro-interfaces where CH_4_ and O_2_ coexist, such as the substrate-air and water-air interfaces, the plant rhizosphere, and within plant tissues (Figure 3; Bonetti et al., 2021). Aerobic CH_4_ oxidation is a chemical process with rapid reaction rates, depending on the concentration of O_2_. Under anaerobic conditions, microbes use electron acceptors other than O_2_ to oxidize CH_4_, including sulfate (sulfate-reduction-dependent anaerobic methane oxidation, SAMO), ${\mathrm{NO}}_{2}^{-}/{\mathrm{NO}}_{3}^{-}$ (nitrite-dependent anaerobic methane oxidation), metal oxides (e.g., Fe^3+^ and Mn^4+^ anaerobic methane oxidation, metal-AOM), and direct interspecies electron transfer (Wegener et al., 2015; Guerrero-Cruz et al., 2021). Among these, SAMO is driven by anaerobic methanotrophs and sulfate-reducing bacteria, while nitrite-dependent AMO is performed by *Candidatus Methylomirabilis oxyfera* and *Candidatus Methanoperedens nitroreducens* (Cui et al., 2015). Additionally, metal-AMO is thermodynamically easier than SAMO (occurring at a 2-to 10-folds faster rate than SAMO) because metal-AMO produces more energy, accelerating the AMO process (Zhang et al., 2022). Mn-AMO has been estimated to reduce total-CH_4_ emissions by 66% (Liu et al., 2020) and Fe-AMO has the potential to decrease CH_4_ emissions by 2-fold if it was to oxidize 10% of CH_4_ worldwide (Egger et al., 2015). Among the different types of CWs, the use of VSSFCWs may be preferable for the reduction of CH_4_ emissions as they have a suitable aerobic/anaerobic interface due to intermittent flooding, which is beneficial for both aerobic oxidation and AOM processes (Zhang et al., 2022).

**Table 2:** Taxonomy and metabolism pathway of aerobic methanotrophs in *Proteobacteria* phylum.

Aerobic methanotrophs types	Class	Order	Family	Genus	Formaldehyde assimilation pathway	Remark
Type I	*γ-Proteobacteria*	*Methylococcales*	*Methylococcaceae*	*Methylomonas*	RuMP pathway	Psychrophiles
				*Methylobacter*		Psychrophiles
				*Methylosarcina*		Thermophiles
				*Methylomicrobium*		/
				*Methyllohalobius*		Haloalkaliphiles
				*Methylosphaera*		/
				*Methylosoma*		Halophiles
				*Methylothermus*		Thermophiles
				*Methylovulum*		Psychrophiles
				*Crenothrix*		/
				*Clonothrix*		/
Type X	*γ-Proteobacteria*	*Methylococcales*	*Methylococcaceae*	*Methylococcus*	RuMP pathway; low levels of enzymes of the serine pathway	Thermophiles
				*Methylocaldum*		Thermophiles
				*Methylogaea*		/
Type II	*a-Proteobacteria*	*Rhizobiales*	*Methylocystaceae*	*Methylocystis*	Serine pathway	Acidophiles
				*Methylosinus*		Acidophiles
			*Beijerinckiaceae*	*Methylocella*		Acidophiles
				*Methylocapsa*		Acidophiles
				*Methyloferula*		Acidophiles
Others	*Verrucomicrobia*	*Methylacidiphilales*	*Methylacidiphilaceae*	*Methylacidiphilum*	A variant of the serine pathway	/

Malyan et al. (2016); Zhu et al. (2016).

Acidophiles means growth at pH of 3.8–5.5; psychrophiles means growth at 5°C–10°C but not above 20°C; thermophiles means growth>45°C; halophiles means growth at 15% NaCl; haloalkaliphiles means growth at 12% NaCl and at pH of 9–11; /means no data.

Methyl coenzyme M reductase (mcrA) and particulate methane monooxygenase (pmoA), are key enzymes in CH_4_ production and oxidation, resulting in their use as phylogenetic biomarkers for methanogens and methanotrophs (Chen et al., 2020b). Methanotrophs are the only known CH_4_ biosinks, oxidizing another component of methanogenesis as carbon sources and producing CO_2_, consuming at least 10% of atmospheric CH_4_ in the process (O'Connor et al., 2010). CH_4_ emissions have been found to positively correlate with the abundance of mcrA (Zhang et al., 2021d), and negatively correlate with the abundance of pmoA (Xu H. et al., 2021). However, no significant relationship was observed between pomA and CH_4_ emissions, suggesting that other factors also mediate their function and activity (Zhang et al., 2021d). The mcrA/pmoA ratio can be used to explore the quantitative relationship between CH_4_ production, oxidation and emissions, with a higher pmoA/mcrA ratio indicating higher CH_4_ oxidation potential and lower CH_4_ emissions (Zhao et al., 2022).

## Environmental factors influencing CH_4_ reduction in CWs

### Temperature

Temperature influences the production and oxidation of CH_4_. The optimum temperature for methanogenesis is typically 35°C–40°C (Ralf, 2007), with low temperatures impairing the activity of methanogens and fermentative bacteria (Barbera et al., 2015) by reducing the rate of organic matter degradation and hence, substrate availability for CH_4_ production (Zhu et al., 2007; Maucieri et al., 2019). CH_4_ production has been found to positively correlate with temperature to some extent under sufficient substrate availability conditions (Bhattacharyya et al., 2013; Zhao et al., 2022). For example, methanogenesis rate at 12°C is significantly lower than that at 30°C (Maucieri et al., 2019). Temperature not only influence microbial activity, but also affect the succession of dominant methanogenic archaea. According to Lu Y. et al. (2015), methanogens were dominated by *Methanosarcinaceae* (utilizing acetate and H_2_/CO_2_ substrates), while as temperature lower, *Methanosaetaceae* dominate the methanogens (using acetate for CH_4_ production). Methanotrophs is temperature non-sensitive species and their optimum temperature is 25°C. CH_4_ can be oxidized either at low to-2°C or at high to 30°C (Zhang et al., 2021a).

As a result of microbial activity influenced by temperature, CWs in warm season can release higher CH_4_ (3.4%–42%) than the cool or cold season (López et al., 2019; Maucieri et al., 2019; Shao et al., 2020). Johansson et al. (2004) found that CH_4_ fluxes in SFCWs were strongly driven by season, fluctuating from-375 mg/m^2^/d to 1739 mg/m^2^/d for the spring to autumn period. Wang et al. (2019) concluded that average CH_4_ emission in summer is 1.7 times higher than in winter, which was also proved by Zhao et al. (2014), D'Acunha and Johnson (2019), and Liu et al. (2019a). Therefore, in order to reduce the release of CH_4_, it is recommended to control the temperature at an appropriate time of higher temperatures in summer.

### Water table position

Water table position determines the degree of anaerobiosis inside CWs. High water tables can exhibit slower rates of atmospheric O_2_ diffusion, creating a larger anoxic zone which is beneficial to CH_4_ production (McInerney and Helton, 2016). Henneberg et al. (2015) compared CH_4_ emissions in CW mesocosms with 0 cm, −10 cm, and −20 cm water tables, showing that CH_4_ emissions were much higher in the 0 cm water table treatment system than the −20 cm system both with and without vegetation. This phenomenon may partly be due to higher levels of CH_4_ dissolution occurring at increased water depths (Zhou et al., 2020). When the water table is below the substrate surface, the CH_4_ produced is oxidized as it travels through the water layer due to diffusion and ebullition, resulting in a reduction in CH_4_ emissions (Bonetti et al., 2021). When the water table is high, CH_4_ emissions are increased as most CH_4_ enters plant root systems in the deeper anaerobic layer, before being transported to the atmosphere through the aerenchyma (Henneberg et al., 2015). A significant positive correlation has been reported between CH_4_ emission rates and water table positions (Liu et al., 2009), with studies also documenting that net CH_4_ fluxes are reduced in systems with a lower water table (Bridgham et al., 2013).

### Redox potential

Redox potential (Eh) is an indicator of the internal O_2_ level in CWs, which determines the activity of microbes and various enzymes, and is closely linked to CH_4_ production and oxidation processes (Liu et al., 2009). Different microbial groups require varying Eh conditions, with aerobic microbes generally requiring an Eh between +300 and + 400 mV. Parthenogenic anaerobic microbes typically have a cut-off Eh of +100 mV, with aerobic respiration occurring at Eh levels above this point and anaerobic respiration occurring at lower Eh levels. Specialized anaerobic bacteria typically require an Eh of-200 to-250 mV. The conversion of organic matter to CH_4_ occurs *via* the general anaerobic digestion pathway, while methanogens at the end of the respiratory chain require a strong reducing environment and very low Eh conditions (optimally-350 mV), with methanogenic processes initiated at Eh conditions < −200 mV (Chen et al., 2020b). The CH_4_ production potential of a system increases with decreasing Eh (Liu et al., 2019b; Shao et al., 2020), and principal component analysis studies have also demonstrated that CH_4_ fluxes are positively correlated with Eh conditions (Liu et al., 2019a).

## The mechanisms and methods of CH_4_ reduction in design of CWs

**Figure 4:**
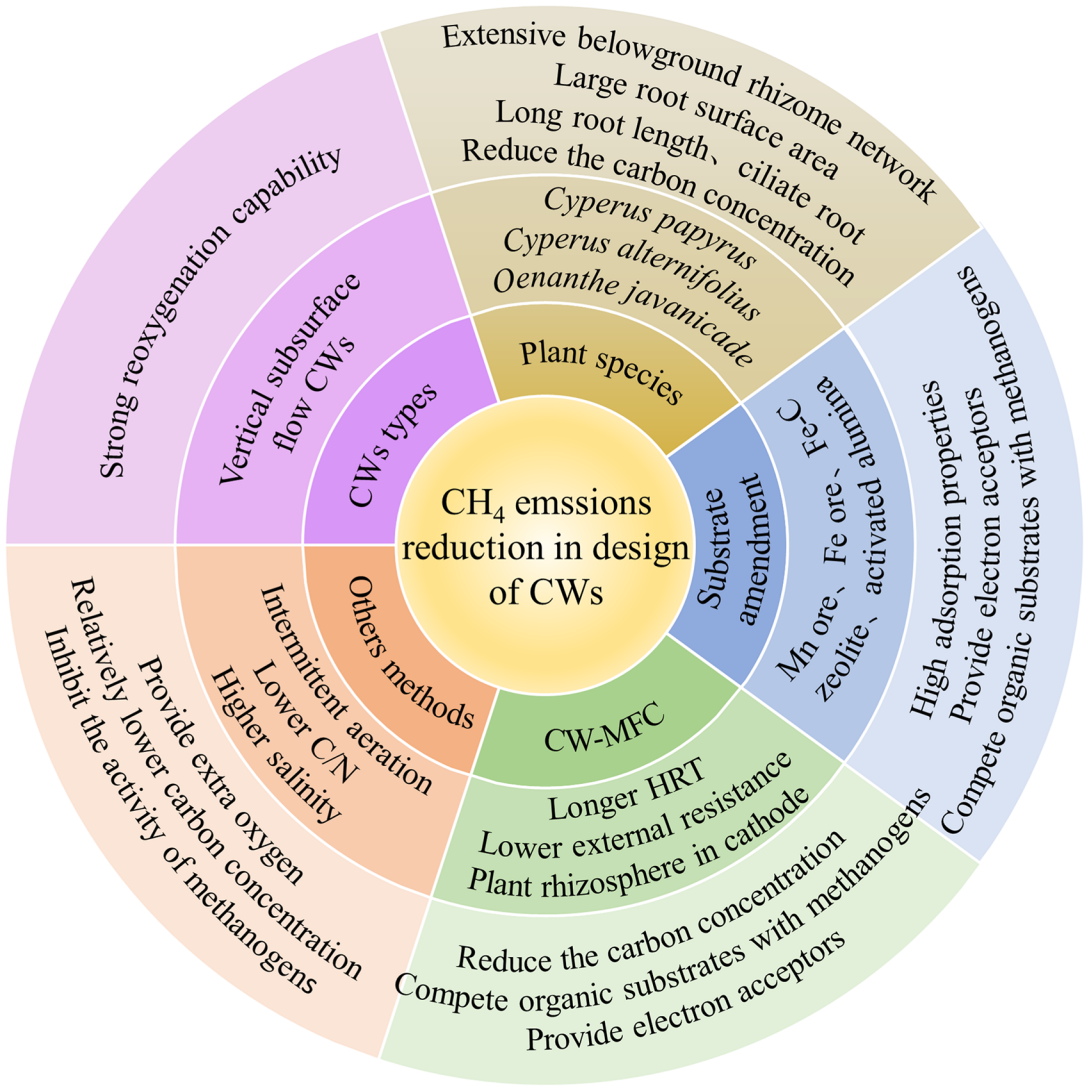
Mechanisms and methods of CH_4_ reduction in design of CWs.

CH_4_ generation, transport, and oxidation are the three main processes that contribute to net CH_4_ emissions from CWs (Bridgham et al., 2013), which are influenced by CWs design. Schemes implemented for the reduction of CH_4_ emissions have mainly depended on the reduction of CH_4_ generation and the promotion of CH_4_ oxidation (Mander et al., 2014). which are affect by CW types, plant species, substrate types, the effect of CW-MFC systems, O_2_ supply, available carbon source, and salinity. Therefore, a comprehensive analysis is required to provide a basis for the effective regulation and operation of CWs, while also actively mitigating GWP contributions (Figure 4; Liu et al., 2017; Maucieri et al., 2017).

### Selection of suitable CW type

CWs are classified as SFCWs, HSSFCWs and VSSFCWs due to their varying structures and characteristics, resulting in significant differences in CH_4_ emission profiles (Zhang et al., 2021a). SFCW systems consist of wastewater flowing over a substrate layer (Maucieri et al., 2017), while VSSFCW systems are gradually infiltrated by wastewater being applied to the surface layer by intermittent feeding, with the wetland bed maintaining an aerobic state with strong reoxygenation capabilities (Liu et al., 2019a). In contrast, wastewater is applied to HSSFCWs through the substrate layer by horizontal percolation in a mixed environment with aerobic, anoxic, and anaerobic degradation pathways, where the bed is submerged for a prolonged period of time causing anoxia (Zhou et al., 2020).

CH_4_ emissions from SSFCWs have been found to be significantly lower than from SFCWs (Table 3). In a survey by VanderZaag et al. (2010), SFCWs were found to emit 2-to 3-fold more CH_4_ as a percentage of carbon removal than SSFCWs. The conditions associated with the highest CH_4_ fluxes from SFCWs and the lowest fluxes from VSSFCWs were studied by Liu et al. (2009). The reason for the observed variation was that SFCWs exhibit very low reoxygenation rates, that are insufficient for the complete oxidation of organic matter and readily create anaerobic conditions that promote CH_4_ release (Søvik et al., 2006), with this effect usually observed in wetland systems that do not have routine harvesting of above-ground plant biomass, providing a continual supply of organic carbon (Hernandez et al., 2018). However, SSFCWs increase the contact time in the aerobic headspace and rhizosphere, which hinders the movement of gases (Zhu et al., 2007), resulting in more CH_4_ oxidation occurring in SSFCWs than in SFCWs and lower CH_4_ fluxes. Liu et al. (2009) also discovered that Eh conditions < −100 mV were primarily found in SFCWs, while no Eh was identified in VSSFCWs, supporting the association between Eh conditions and the high CH_4_ fluxes observed in SFCWs.

**Table 3:** CH_4_ emissions in different types of CWs.

CW types	Substrate types	Vegetation types	CH_4_ fluxes (mg/m^2^/h)	Wastewater types	Reference
SF	Sand	*Phragmites australis*	0.09	Domestic Wastewater	Zhu et al. (2007)
SSF		*Phragmites australis*	0.16		
SF		Unvegetated	0.37		
SSF		Unvegetated	0.14		
SF	Pea-stone and gravel	*Typha latifolia*	9.3	Dairy farm wastewater	VanderZaag et al. (2010)
SSF			4.9		
SF	Sand	*Phragmites australis*	61.67	Municipal Wastewater	Gui et al., 2007
HSSF			8.18		
VSSF			3.23		
SF	Sand	*Phragmites australis*	26	Domestic Wastewater	Liu et al. (2009)
HSSF			5.4		
VSSF			1.7		
SF	Cobble	*Cyperus alternifolius*	0.64	River Wastewater	Zhang et al. (2021a)
HSSF	Gravel	*Cyperus alternifolius*	0.15		
VSSF	Gravel	*Cyperus alternifolius*	0.42		
HSSF	Gravel	*Cyperus papyrus*	22.70 ± 1.9	Municipal Wastewater	Bateganya et al. (2015)
HSSF	Gravel	Unvegetated	38.30 ± 3.3		
VSSF	Sand and gravel aggregates	*Cyperus papyrus*	3.30 ± 0.4		
VSSF	Sand and gravel aggregates	Unvegetated	13.60 ± 1.4		
HSSF	Gravel	*Phragmites australis*	31.8	Piggery farm wastewater	Zhou et al. (2020)
VSSF	Gravel	*Phragmites australis*	6.6		

CH_4_ fluxes from HSSFCWs were found to be higher than those from VSSFCWs (Table 3). For example, Bateganya et al. (2015) found that HSSFCWs emit more CH_4_ than VSSFCWs, irrespective of plant growth. Zhou et al. (2020) designed a hybrid CW system that generated about 4.8-fold lower CH_4_ fluxes in the VSSF bed section than the HSSF bed section. The reason for this difference may be attributed to VSSFCWs having efficient O_2_ transport, while HSSFCWs contain anoxic-anaerobic conditions (Mander et al., 2008), exhibiting negative Eh levels (−100 mV to-500 mV) and low dissolved oxygen (DO) concentrations (< 2 mg/L; López et al., 2015). In contrast, the VSSF system was found to be higher than the HSSF system in a study by Zhang et al. (2021a), due to the potential for CH_4_ emissions to be affected by competition between various methanogens. A life cycle assessment also concluded that the environmental impact of CH_4_ emissions from VSSFCWs was half or less than those from HSSFCWs (Fuchs et al., 2011).

CWs are increasingly being designed with composite structures that are more valid and robust for the treatment of a wide range of wastewater types (Liu et al., 2019a), However, these composite CW systems often have a negative effect on CH_4_ fluxes (Zhou et al., 2020). For example, a monitoring study found that CH_4_ emissions from VSSF-HSSF-SF CW was higher than from both VSSFCW and SFCW (Liu et al., 2009). Liu et al. (2019a,b) and Liu et al. (2018) designed integrated VSSFCWs that consist of alternating multifunctional layers of aerobic-anoxic-anaerobic-anoxic-aerobic conditions, leading to the accumulation of methanogens and increased CH_4_ emissions (Zhang et al., 2021a). Waste resource conservation and reducing environmental influence have become priorities in sustainable engineering design. Therefore, the small occupation area of VSSFCWs, along with their high treatment efficiency and relatively low level of CH_4_ fluxes, make VSSFCW systems more extensively used, and a more CH_4_ flux can be reduced *via* an enhanced O_2_ transfer approach (Liu et al., 2019b).

### Plant species selection

The relationships between CH_4_ emissions and plant occurrence and diversity in CWs remain unknown (Han et al., 2019), as plants are able to both produce CH_4_ independently, and mediate or influence CH_4_ emission pathways. For example, organic matter and root exudates (such as sugars and amino acids) synthesized by plants *via* photosynthesis, can provide electron donors (Chen et al., 2020c; Liu et al., 2022), while root exudates also release degradable carbon which increases the number of methanogens and methanotrophs (Zhang et al., 2018b). Furthermore, plant root-secreted O_2_ can provide electron acceptors (Kasak et al., 2020), with the intensity of O_2_ secretion varying depending on the plant species, biomass, temperature, O_2_ concentration, and photointensity conditions (Feng et al., 2022). In typical VSSFCWs, O_2_ released by plant roots can provide approximately 0.43–1.12% of the biochemical oxygen demand (Zhang et al., 2014). The rhizosphere is a crucial zone for the production and oxidation of CH_4_, with plant species composition, diversity, and planting density affecting the release of CH_4_ (Chen et al., 2020c).

It has been observed that vegetation-covered CWs produce less CH_4_ than those without vegetative cover (Zhu et al., 2007; Bateganya et al., 2015). However, contradictory results have been reported by Silvey et al. (2019), with this variation potentially due to plant species variation. Chen et al. (2020c) reported that planting *Cyperus alternifolius* resulted in CWs having lower CH_4_ fluxes than unvegetated CWs, while planting *Phragmites australis* and *Canna indica* had the opposite effect, highlighting the varying effect of different species on CH_4_ fluxes. Firstly, some plants have been found to suppress CH_4_ fluxes (Table 4). A monitoring study by Bateganya et al. (2015) found that planting *Cyperus papyrus* was more effective for the suppression of CH_4_ fluxes regardless of the CW types, due to the extensive belowground rhizome network facilitating O_2_ transfer (Xu et al., 2019; Zhang et al., 2021a). *Cyperus alternifolius* is a ciliate with large root surface areas and root lengths of approximately 20 cm, providing them with the capacity to reach the bottom of CWs and release high amounts of root O_2_, leading to a more extensive aerobic environment (Chen et al., 2020c). The presence of *Oenanthe javanicade* has been shown to reduce the carbon concentration in wastewater, minimizing the production of CH_4_ (Zhao et al., 2016; Han et al., 2017). In contrast, some plant species enhance CH_4_ emissions (Table 4; Du et al., 2018; Maucieri et al., 2019). *Juncus effusus* has a significant capacity to transport CH_4_ (Henneberg et al., 2015), and *Rumex japonicus* possesses a high root biomass capable of secreting low molecular weight substances (Zhao et al., 2016), accelerating microbial activity and increasing CH_4_ emissions (Du et al., 2018). Overall, the contribution of plants to CH_4_ emissions remains unclear. For example, Ph*ragmites australis* has an extensive rhizome system that typically penetrates substrate depths of 0.6–1.0 m, and has been reported to effectively reduce CH_4_ fluxes (Xu et al., 2019), while other studies have reported that *Phragmites australis* possesses highly developed aerenchyma that allow more efficient gas transfer and increase CH_4_ emissions (Sheng et al., 2015; Chen et al., 2020c). Moreover, some studies have proposed that certain plant species has no overall impact on CW CH_4_ emissions (Han et al., 2019; Maucieri et al., 2019). Instead, plant characteristics such as root porosity and belowground biomass can regulate microbial community competition, O_2_ transfer efficiency to the root system, and carbon source inputs, leading to varying levels of CH_4_ productivity in different species (Maucieri et al., 2017; Zhang et al., 2018b). The average CH_4_ fluxes of submerged plants are generally lower than those of emergent plants (Niu et al., 2015; Zhang et al., 2018a). O_2_ and carbon inputs from emergent plants can significantly affect the methanogenic community structure and methanogenic pathways, while submerged plants are primarily subject to regulation by DO and nitrogen levels (Zhang et al., 2018a). Furthermore, in contrast to the comparatively less-rigid forb species, some structurally rigid graminoids exhibit larger aerenchyma, which increases their ability to transport O_2_ between the roots. Mesocosms containing *Asclepias incaranta* were found to have average CH_4_ fluxes that were 8-fold higher than those of mesocosms containing *Alisma triviale* (Silvey et al., 2019). Therefore, the selection of suitable plant species composition is essential to minimize CH_4_ emissions from CWs.

**Table 4:** CH_4_ emissions in CWs planted different vegetation.

Vegetation types	CWs types	Substrate types	CH_4_ fluxes (μg/m^2^/h)	Wastewater types	References
Emergent vegetation					
*Phragmites australis*	SFCW	/	2,640	Agricultural wastewater	de Klein and van der Werf (2014)
*Phragmites australis*			1820		
Unvegetated			7,430		
Unvegetated			19,810		
*Cyperus papyrus*	HSSFCW	Gravel	22,700 ± 1900	Municipal wastewater	Bateganya et al. (2015)
Unvegetated			38,300 ± 3,300		
*Cyperus papyrus*		Sand and gravel	3,300 ± 400		
Unvegetated			13,600 ± 1,400		
*Juncus effusus*	SFCW	/	20,380 ± 1930	Sewage treatment water	Ström et al. (2006)
*Phragmites australis*			13,880 ± 3,190		
*Typha latifolia*			9,380 ± 1990		
Unvegetated			200 ± 2,580		
*Arundo domax*	HSSFCW	Gravel	25,170	Municipal wastewater	Maucieri et al. (2014)
*Phragmites australis*			21,160		
Unvegetated			18,100		
*Phragmites australis*	HSSFCW	Gravel	20,220 ± 6,700	Municipal wastewater	López et al. (2015)
*Schoenoplectus Californicus*			18,120 ± 1,130		
*Rumex japonicus*	VSSFCW	Sand	310	Synthetic wastewater	Niu et al. (2015)
*Oenanthe hookeri*			200		
*Phalaris arundinacea*			290		
*Juncus effusus*			190		
Unvegetated			140		
*Rumex japonicus*	/	Fine sand	285 ± 102.5	Synthetic wastewater	Zhao et al. (2016)
*Oenanthe javanica*			21.67 ± 58.33		
*Phalaris arundinacea*			140 ± 117.08		
*Juncus effuses*			154.58 ± 114.58		
*Rumex japonicus*	/	Coarse sand	245.83 ± 8.75		
*Oenanthe javanica*			256.25 ± 8.33		
*Phalaris arundinacea*			250 ± 7.5		
*Juncus effuses*			240 ± 6.25		
*Rumex japonicus*	VSSFCW	Sand	285.8	Synthetic wastewater	Han et al. (2017)
*Oenanthe javanica*			232.08		
*Phalaris arundinacea*			242.92		
*Juncus effuses*			210.83		
*Typha orientalis*	VSSFCW	Sand and gravel	10,100	River wastewater	Zhang et al. (2018b)
*Cyperus alternifolius*			15,100		
*Arundo domax*			12,500		
*Iris pseudacorus*			19,400		
*Thalia dealbata*			7,100		
*Phragmites australis*	HSSFCW	Gravel	30,000	Municipal wastewater	Maucieri et al. (2019)
*Arundo donax*			34,000		
*Chrysopogon zizanioides*			45,000		
*Miscanthus × giganteus*			59,000		
Unvegetated			25,000		
*Lolium perenne*	VSSFCW	Sand	0.011	Synthetic wastewater	Han et al. (2019)
*Cichorium intybus*			0.025		
*Medicago sativa*			0.033		
*Rumex japonicus*			0.014		
*Canna indica*	SSFCW	Gravel	13.66 ± 24	Synthetic wastewater	Chen et al. (2020c)
*Cyperus alternifolius*			−34.60 ± 8.12		
*Phragmites australis*			21.88 ± 2.51		
Unvegetated			−5.32 ± 7.14		
Submerged vegetation					
*Potamogeton crispus*	/	/	5,700	Municipal wastewater	Zhang et al. (2018a)
*Myriophyllum spicatum*			1,600		
*Hydrilla verticillata*			4,000		

/ means no data.

Plant species diversity has been widely studied in recent years, and its contribution to CH_4_ emissions has increasingly being investigated. Several studies have observed a positive correlation between CH_4_ emissions and plant species diversity (Du et al., 2018), as high species diversity increases carbon source available and promotes CH_4_ emissions (Zhang et al., 2012). Some studies have also found that plant species diversity have no significant influence on CH_4_ emissions (Zhao et al., 2016; Han et al., 2019), as in high ammonium environments, denitrification has a higher capacity to produce thermodynamic processes than methanogenesis (Chen et al., 2020c). In addition, denitrifying bacteria have a competitive organic substrate advantage, limiting CH_4_ formation (Zhang et al., 2018a), in which high ammonium loading and plant biomass can perform CH_4_ offsetting functions, leaving CH_4_ emissions unchanged overall (Han et al., 2019). Moreover, plant density has been reported to have no influence on CH_4_ emissions (Hernandez et al., 2018). In conclusion, species characteristics remain a critical driver although more research is required in this field.

The contribution of plant harvesting to CH_4_ emissions is also significant (Feng et al., 2022). Non-harvested wetland plants can promote methanotrophic CH_4_ consumption by transporting O_2_ to the roots and substrate through aerenchyma (Zhu et al., 2007). However, the dying plant biomass provides an abundant source of bioenergy and promotes methanogenesis, with the potential to generate 10–40% of annual atmospheric CH_4_ emissions worldwide (Juutinen et al., 2003; Keppler et al., 2006), highlighting the need for plant harvesting to be carefully managed. The time and manner of harvesting of aboveground macrophytes also affects CH_4_ emissions from CWs (Kasak et al., 2020). Ensuring that harvesting occurs at the end of the growing season (i.e., before nutrient transfer to belowground plant structures) can significantly reduce CH_4_ emissions. However, biomass harvesting during peak periods of plant growth and soil microbial activity has been shown to significantly enhance CH_4_ emissions (Barbera et al., 2015), due to the rapid release of CH_4_ accumulated in the vascular system of plant stalks (Kasak et al., 2020). Therefore, effective planning to optimize harvesting time and method can effectively reduce CH_4_ emissions from managed wetlands, and thus enhance their multiple ecological benefits.

## Substrate amendment

The substrate forms the backbone of CWs, providing support for the growth of plants and microbes (Ji et al., 2021). Recently, novel substrate amendment schemes have been applied in CWs, with the addition of substances such as biochar, iron oxides, manganese oxides, zeolite, walnut shell, activated alumina, and ferric-carbon, which are gradually displacing traditional substrates (such as sand, gravel, and ceramsite) and improving the treatment efficiency of CWs. Some reviews have focused on the impact of substrate on CH_4_ emission reduction. However, none of these have emphasized the role of enhanced substrates compared to conventional substrates (Yu et al., 2022; Zhao et al., 2022). Therefore, in order to achieve sustainable and low-environmental impact CW operations, we summarized multifarious functional substrates used in CWs for enhancing the CH_4_ emissions reduction.

### Carbon-rich substrate types

Biochar is an organic carbon-enriched product that is considered a promising alternative substrate (Zhuang et al., 2022). Ji et al. (2021) showed that biochar-based CWs contained a higher pmoA/mcrA ratio than none-biochar CWs, with the addition of biochar having an inhibitory effect on CH_4_ fluxes, possibly due to biochar promoting the secretion of O_2_ from plant roots, increasing CH_4_ oxidation (Ji et al., 2020). However, Chen et al. (2020a) and Guo et al. (2020) proposed contrasting conclusions, finding that CH_4_ fluxes were consistently higher in biochar-added CWs than CWs without added biochar. This may be due to biochar enhancing direct interspecies electron transfer between methanogens and *Geobacteraceae,* while also providing organic matter to methanogens, resulting in the stimulation of CH_4_ emissions (Liu et al., 2012). Overall, the influences of biochar on CH_4_ emissions remains unclear (Table 5), with the reported differences primarily caused by variations in the raw biochar materials, operating conditions, and the properties of the microbial community within the system (Chen et al., 2020a), highlighting the need for further research to determine the role of biochar in regulating CH_4_ emissions (Zhuang et al., 2022). Walnut shell is also loaded with high concentrations of organic matter. Xu G. et al. (2021) showed that CH_4_ emissions from walnut shell substrate were 14.8-fold higher than from the control substrate, due to the release of large amounts of degradable organic carbon from walnut shell, resulting in high CH_4_ fluxes.

**Table 5:** CH_4_ emissions in CWs with different substrates.

Substrate types	CW types	Vegetation	CH_4_ fluxes (mg/m^2^/h)	Main operation conditions	References
Biochar and ceramsite^a^	SSFCW	*Lythrum salicaria*	0.17 ± 0.11	Non-aeration	Ji et al. (2020)
Ceramsite			0.25 ± 0.19	Non-aeration	
Biochar and ceramsite			−0.058 ± 0.077	Aeration	
Biochar and ceramsite			0.12 ± 0.14	Tidal flow	
Biochar and gravel	/	*Typha latifolia*	0.2192–0.477.0	Non-aeration	Guo et al. (2020)
Gravel			0.1274–0.2708	Non-aeration	
Biochar and coarse gravel	SSFCW	*Canna indica*	0.023	Non-aeration	Chen et al. (2020a)
Coarse gravel			0.003	Non-aeration	
Biochar and coarse gravel	SFCW		0.03	Non-aeration	
Coarse gravel			−0.029	Non-aeration	
Ceramsite	SSFCW	*Lythrum salicaria*	0.24 ± 0.01	Non-aeration	Ji et al. (2021)
Biochar and ceramsite			0.08 ± 0.08	Non-aeration	
Biochar and ceramsite			0.03 ± 0.03	Aeration	
Gravel	VSSFCW	*Cyperus alternifolius*	229.17 ± 10	Non-aeration	Liu et al. (2020)
Mn ore			125.42 ± 15.83		
Gravel and sand	VSSFCW	*Iris pseudacorus*	17.08	Non-aeration	Xu G. et al. (2021)
Mn ore, gravel and sand			2.00		
Walnut shell, gravel and sand			252.30		
Activated alumina, gravel and sand			6.43		
Quartz sand	VSSFCW	/	0.059–0.061	Non-aeration	Cheng et al. (2021a)
Iron ore and quartz sand			0.048–0.051		
Gravel and quartz sand	VSSFCW	*Yellow calamus*	0.06	Non-aeration	Cheng et al. (2021b)
Iron ore, gravel, and quartz sand			0.05		
Mn ore, gravel, and quartz sand			0		
Gravel	VSSFCW	*Phragmite australis*	31.8	Non-aeration	Zhou et al. (2020)
Zeolite			16.6		
Gravel	VSSFCW	*Acorus calamus*	0.41 ± 0.07	Aeration	Zhao et al. (2022)
Zeolite and gravel			0.20 ± 0.03		
Fe-C and gravel			0.31 ± 0.04		
Fe-C, zeolite, and gravel			0.21 ± 0.03		

a means the mix of two or three types of substrates.

/means no data.

### Electron-exchange substrate types

Highly crystalline iron oxides and manganese oxides are electron-exchange substrates that are abundant and readily available, making them highly suitable for use as CW substrate materials (Zhang et al., 2021b; Yu et al., 2022). Cheng et al. (2021a) reported that CWs using iron oxide substrates emitted less CH_4_, finding that iron oxide increased CH_4_ emissions by promoting electron transfer between *Geobacter* and methanogens, while also directly inhibiting the activity of methanogens and some enzymes involved in CO_2_ reduction, and promoting the AOM process under the influence of dissimilated metal-reducing bacteria, ultimately resulting in a reduction in CH_4_ emissions overall (Cheng et al., 2021b). Mn ore substrates have been found to reduce CH_4_ emissions from CWs (Liu et al., 2020). In addition, Cheng et al. (2021b) observed that both Mn ore and iron ore substrates inhibited CH_4_ emissions, with Mn ore reported to be more effective due to the fact that Mn ore promotes AOM processes mainly by competing for organic substrates and providing electron acceptors, almost completely inhibiting CH_4_ production (Xu G. et al., 2021). However, the use of iron oxide as a substrate has more complex implications, such as different forms and valences of iron affecting CH_4_ production (Cheng et al., 2021a).

### Adsorption substrate types

Fe-C is widely used as a substrate in wastewater treatment, utilizing chemistry-coupled biological processes for the removal of pollutants (Dong et al., 2020). Zeolite, a common silicate mineral, is considered to be a high-performance gas adsorption material, due to its relatively well-developed pore network and skeletal configuration (Wang Y. et al., 2020), with well characterized CH_4_ adsorption, storage, and oxidation capabilities (Wang H. et al., 2020; Zhou et al., 2020). Zhao et al. (2022) monitored CH_4_ emissions from laboratory-scale CWs containing different substrates, showing that systems utilizing Fe-C and zeolite as substrates all exhibited lower average CH_4_ fluxes than the control groups (Table 5), as Fe-C can compete with methanogens for substrate in the presence of iron-reducing bacteria, inhibiting the production of CH_4_. In addition, Fe^3+^ has a high Eh as an electron acceptor (Bond and Lovley, 2002), and the larger surface area of activated carbon facilitates biofilm generation, promoting CH_4_ oxidation. Zeolite incorporation into the substrate has been found to significantly reduce CH_4_ emissions from CWs. Zhou et al. (2020) discovered that CWs containing zeolite substrate exhibited reduced CH_4_ fluxes by about 2-fold compared to those of gravel substrate CWs, due to the porous structure of zeolite improving local atmospheric DO concentrations and reducing methanogen activity. The results of these studies are further supported by the observation that Fe-C and zeolite substrates contained a reduced abundance of the functional gene mcrA and a significantly increased abundance of pmoA (Zhao et al., 2022). Activated alumina is also a powerful adsorption substrate, as shown in a study by Xu G. et al. (2021) in which activated alumina significantly reduced CH_4_ emissions from CWs due to its strong adsorption capacity, leading to reduction in organic matter content and inhibiting methanogenic microbial activity (Alvarez and Cervantes, 2012). In addition, sand, ceramsite and gravel have also been utilized as adsorption fillers (Table 5; Wang Y. et al., 2020), but they have been gradually replaced due to poor CH_4_ adsorption capacities (Ji et al., 2020; Cheng et al., 2021a; Zhao et al., 2022). With a survey of CWs finding that mcrA and pmoA were not detectable in all gravel substrates (Chen et al., 2020c), which was attributed to the fact that methanogens and methanotrophs do not easily attach to gravel so it is gradually replaced.

## CW-coupled MFC systems

MFCs are a low-environmental impact energy utilization technology (Wang et al., 2019; Zhang et al., 2021d). CWs (especially VSSFCWs) have unique water quality conditions with substantial redox gradients across their vertical profile, with DO and Eh levels increasing from the subsurface to the surface zones (Maucieri et al., 2017). As CWs and MFC systems function under similar conditions, it is feasible to control CH_4_ emissions using CW-coupled MFC systems (Zhang et al., 2021b,c). Methanogens and electrogenic bacteria require similar living conditions, such as an anaerobic environment and low Eh, resulting in competition for the substrate at CW-MFC anodes, which may inhibit the power generation performance of the CW-MFC (Liu et al., 2017; Zhang K. et al., 2020). A comprehensive understanding of the competitive mechanisms between methanogens and electrogens would help maximize the potential advantages of CW-MFC systems for efficient biopower generation and the reduction of CH_4_ emissions (Zhang et al., 2021e), along with determining the influences of circuit condition, plant type, external resistance, substrate type, and hydraulic retention time (HRT).

### Open/closed circuit in CW-MFC

Open or closed circuit systems are one of the essential factors affecting CH_4_ fluxes from CW-MFCs. Liu et al. (2022) found that the CH_4_ emissions from non-planted CW-MFCs, were about 0.21 ± 0.01 mg/m^2^/h higher in open-circuit systems than in closed-circuit systems, while in the planted open-circuit system the CH_4_ emissions were 0.46 ± 0.02 mg/m^2^/h higher than in the closed-circuit planted system. A study by Xu et al. (2021b) yielded CH_4_ fluxes of 6.37–7.28 mg/m^2^/h and 7.43–8.36 mg/m^2^/h for closed and open circuit CW-MFC systems, respectively. Zhang et al. (2021e) noted that running a MFC in CWs can suppress a third of all CH_4_ emissions. These studies show that CW-MFCs can effectively reduce CH_4_ emissions from CWs, with similar findings also reported by Zhang K. et al. (2020) and Zhang et al. (2021c), with the main contributory factor being the bioanode. In anaerobic environments, methanogens are dominant in CWs due to the absence of current transmission, resulting in an increase in the production of CH_4_ (Zhang et al., 2021d). In contrast, because organic matter is more readily available for electrochemically active bacteria (EAB) in closed-circuit CW-MFC systems, electrical stimulation of EAB growth results in a current that inhibits methanogens (Liu et al., 2022). In addition, electrons from the anode may compete with methanogens as the anode layer of the CW-MFC has a higher Eh, allowing electron-producing bacteria to capture electrons more easily than methanogens, leading to a reduction in CH_4_ emissions (Zhang et al., 2021e).

### Plant rhizosphere location

CH_4_ emissions are significantly affected by plants in CW-MFC systems. Recently, an increasing number of studies have demonstrated that plant root location has an influential effect on CH_4_ emissions in CW-MFC systems (Liu et al., 2017). When the plant rhizosphere is in the cathode layer, electron acceptors provided by root-secreted O_2_ favor cathode reactions and power generation (Zhang et al., 2021e). Since CW-MFC anodes are typically in an anoxic state rather than an anaerobic state, the small amount of O_2_ secreted by plant roots has little effect on the O_2_ levels in the anode environment (Zhang et al., 2021d). Therefore, plant rhizospheres in the anodic zone can provide photosynthetic organic matter as an alternative energy source, increasing CH_4_ emissions. Zhang et al. (2021e) reported that CW-MFC systems with rhizospheres in the cathode zone emit less total-CH_4_ (29.21 mg/m^2^/d) than those with rhizospheres at the anode (33.01 mg/m^2^/d). Zhang et al. (2021d) observed that growing *Typha orientalis* and *Cyperus alternifolius* in the cathode zone of a reactor, resulted in lower CH_4_ emissions than when grown at the anode. Zhang et al. (2021c) reported a similar conclusion, with methanogens becoming more active as the organic matter content of the rhizosphere increases, resulting in an increase in CH_4_ emissions. Additionally, the O_2_ provided by plant roots has been found to have less of an impact on lowering CH_4_ emissions than the organic matter in the root system (Lu L. et al., 2015).

The presence of plants and the selected plant species also influence CH_4_ emissions in CW-MFC systems. Liu et al. (2022) reported that CH_4_ emissions increased by 0.48 ± 0.02 mg/m^2^/h in closed-circuit CW systems with plants, compared to the non-planted group. A similar conclusion was reached by Xu et al. (2021b), with a 21.79% reduction in average CH_4_ emissions from non-planted reactors, as compared to those with plants. Zhang et al. (2021d) reported lower CH_4_ emissions in systems with anode grown *Typha orientalis* (3.9 mg/m^2^/h), than with anode grown *Cyperus alternifolius* (4.5 mg/m^2^/h). A study comparing the effects of three plant species, *Typha orientalis*, *Thalia dealbata*, and *Cyperus alternifolius*, found that CH_4_ emissions from the CW-MFC were highest in the *Typha orientalis* system, and lowest in the *Cyperus alternifolius* system (Zhang K. et al., 2020). These results emphasize the importance of further research to determine the effect of different plant species on CH_4_ emissions from CW-MFC systems.

The important role that plants play in CH_4_ emissions from CW-MFC systems is becoming increasingly apparent, with both the plant species and root location influencing CH_4_ emissions, although root location appears to have a more pronounced effect (Zhang et al., 2021d). Therefore, focusing on plant selection to ensure that plants with inhibiting methanogenesis or poor CH_4_ transport are utilized, with their roots located in the cathodic zone, has been shown to be more advantageous for the sustainable development of wastewater treatment systems, in terms of reactor power generation and CH_4_ reduction.

### External resistance

The long-term application of external resistance in CW-MFC systems affects the microbial community structure and biochemical metabolism in the anode biofilm (Zhang et al., 2021c). Liu et al. (2022) observed that an external resistance of 1,000 Ω leads to an increase in CH_4_ emissions by 0.67 ± 0.01 mg/m^2^/h compared to a 100 Ω resistance. Wang et al. (2019) found that CH_4_ emissions tended to increase with the addition of external resistances >500 Ω. This effect occurs due to variation in microbial metabolic activities, electron transfer rates and substrate utilization kinetics, under varying external resistance conditions (Liu et al., 2022). Higher external resistance loads slow down the flow of electrons to and from EAB, which encourages methanogens to consume more substrate and increases CH_4_ emissions (Liu et al., 2017). However, it has also been found that insufficiently low external resistances lead to rapid electron flow rates, which are not conducive to the sustainable utilization of CW-MFC systems, and eventually leads to a decline in power generation (Nikhil et al., 2018). Therefore, a suitable relatively low external resistance should be applied, ensuring that CW-MFC systems generate maximum levels of power, while releasing minimum CH_4_ (Liu et al., 2022).

### Additional influencing factors

*Substrate*. CH_4_ emissions in CW-MFC systems are also driven by substrate type. A Mn CW-MFC system was shown to generate lower CH_4_ emissions (53.44–66.64 mg/m^2^/h) than a clinopyrite CW-MFC system (62.69–88.02 mg/m^2^/h; Zhang et al., 2021b). Furthermore, Xu et al. (2021b) observed a 25.42% reduction in average CH_4_ emissions from Mn-based reactors compared to graphite granule-based reactors. This may be due to the occurrence of Mn-driven Mn-AOM lowering CH_4_ emissions, while dissimilatory metal reduction processes encourage competition between EABs and methanogens, as well as increasing the growth of EABs on Mn ore anodes, further inhibiting the growth of methanogens (Li et al., 2019; Zhang et al., 2021b, 2022). Therefore, different substrate materials have varying electron acceptor functions and significantly affect CH_4_ emissions, with Mn exhibiting high prospects as a substrate for CH_4_ emissions reduction.

*HRT*. Due to the relatively high organic load in CW-MFCs, there is a positive correlation between CH_4_ emissions and HRT. Lower HRTs result in a higher load and increased CH_4_ emissions (Zhang et al., 2021e). It was observed that when the HRT in plant systems increased, CH_4_ fluxes tended to reduce, with higher organic matter contents stimulating the activity of inactive microorganisms, consuming DO, and promoting the growth of methanogens (Wang et al., 2019). HRT is an extremely vital operating parameter for conventional wetlands (Zhang K. et al., 2020). A longer HRT can prolong the contact time between microorganisms and wastewater (Zhang et al., 2021d), ensuring the effective removal of organic matter as well as the suppression of CH_4_ emissions. Finally, it has been found that CW-MFC systems have a stronger CH_4_ reduction effect when operated in continuous flow mode than in batch mode (Wang et al., 2019).

## Other methods

### Oxygen supply strategy

Shortage of DO in CWs due to prolonged saturation and rapid microbial metabolism is a major limitation to the removal of organic matter from conventional CWs (Wang et al., 2022). Intermittent aeration and tidal flow are considered to be the most effective oxygenation strategies to directly influence CH_4_ emissions, allowing the manipulation of DO conditions in CWs (Table 5; Ji et al., 2020), providing additional O_2_ for the inhibition of CH_4_ biochemical processes and the acceleration of CH_4_ oxidation, reducing CH_4_ fluxes (Ji et al., 2021). D'Acunha and Johnson (2019) found that CH_4_ fluxes could be reduced by 60.7% using intermittent microaeration under optimal aeration conditions. Furthermore, Zhao et al. (2022) observed increased CH_4_ fluxes in aerated sections of CWs compared to non-aerated sections, which was attributed to CH_4_ production primarily occurring in non-aerated sections, while CH_4_ emissions were enhanced by agitation and blowing in aerated sections, with the change in emissions consistent with the aeration rate and dissolved CH_4_ concentration. Ji et al. (2020) showed that only a slight decrease in CH_4_ fluxes occurred under tidal flow oxygenation conditions, although Ji et al. (2021) concluded that the total CH_4_ fluxes were slightly higher due to the loss of large amounts of CH_4_ from the empty substrate after drainage. However, the use of intermittent aeration increases operational costs and energy input requirements, making it more suitable for use in CWs in relatively concentrated communities or limited land areas. In contrast, the tidal flow process involves no significant additional costs or maintenance expenses.

### Carbon source supplement

The available carbon sources in CWs can be supplied internally or externally. Internal carbon sources mainly include plant roots or microbial secretions, plant deadfall, organic matter decomposition, and substrates (Liu et al., 2019a), while external carbon sources include biodegradable carbon, natural plant material, and soluble carbon from natural organic matter (Liu et al., 2019b). To balance the nutritional ratio in CWs, additional carbon sources must be provided in some CWs, directly affecting CH_4_ emissions (Chen et al., 2020b). C/N ratios represent the relative amount of carbon and nitrogen available in the wastewater, with different C/N ratios promoting or inhibiting microbial activity (Guo et al., 2020). Yan et al. (2012) found that CH_4_ fluxes in CWs with C/N ratios of 2.5, 5, and 10 were 1.36 ± 0.09 mg/m^2^/h, 2.02 ± 0.07 mg/m^2^/h, and 2.34 ± 0.15 mg/m^2^/h, respectively, showing that CH_4_ fluxes were lowest with low C/N ratio conditions. Corbella and Puigagut (2015) also reported a positive correlation between CH_4_ emissions and influent C/N conditions, which may be attributed to increases in C/N causing rapid O_2_ consumption, resulting in a lower Eh and higher CH_4_ production (Guo et al., 2020). However, Chen et al. (2020b) analyzed CWs with influent C/N ratios of 0, 5, 10, 15, and 20, showing that CH_4_ emissions did not differ significantly, reaching a minimum value of −42.49 mg/m^2^/h at a C/N ratio of 10. Zhao et al. (2014) combined SSFCWs with an earthworm eco-filter and found that CH_4_ emissions increased in accordance with the influent C/N ratio regardless of the sequence of treatment, as the earthworm activity greatly increased the permeability of the SSFCW. Therefore, lower C/N ratio conditions are more favorable for CH_4_ emissions reduction. Liu et al. (2019a) observed that the addition of urea as an external carbon source at concentrations of 0, 12.1, 30, 45, 61, and 80 mmol/L, resulted in a trend of increasing and then decreasing CH_4_ emissions, achieving the lowest emission level with a urea concentration of 80 mmol/L. Similar results were obtained by Liu et al. (2019b) using ethanol as an external carbon source, with results showing that Eh values after the addition of ethanol (except for the highest ethanol concentration of 32 mmol/L) were higher than the control groups, resulting in more CH_4_ emissions. Excessive carbon source concentration increases the water purification load. Therefore, when external carbon sources are provided, the dosage should be adjusted to ensure optimal water purification performance with CH_4_ emission control.

### Salinity control

With increasing levels of water scarcity, desalination, or the direct utilization of seawater in coastal areas is becoming increasingly common, generating large quantities of saline wastewater. CWs are increasingly being used for the treatment of saline wastewater in response to different regional treatment strategies (Shao et al., 2020). The salinity of the influent affects not only the growth of wetland plants but also the structure, abundance and activity of microbial communities (Sheng et al., 2015; Xiao et al., 2016). According to Shao et al. (2020), CH_4_ emissions were higher at salinities of 0 and 0.5%, continually decreasing with increasing salinity, with a substantial decrease occurring at 1.0%. Sheng et al. (2015) also observed that CH_4_ emissions decreased significantly with increasing salinity due to inhibition of methanogen growth and activity (Xiao et al., 2016). In addition, salinity has been repeatedly shown to inhibit CH_4_ emissions in tidal wetlands, due to competition between sulfate and methanogens for substrates in tidal waters (Poffenbarger et al., 2011; Marton et al., 2012). In summary, the effect of salinity on CH_4_ fluxes in CWs is pronounced, with emissions promoted by low salinity and inhibited by high salinity conditions.

## Conclusions and perspectives

Global climate change is a complex phenomenon. CWs are a highly productive green technology for surface source pollution control, which are designed to treat wastewater using the natural processes of plants, substrates and microorganisms, utilizing carbon cycling processes that are an essential part of the global system, generating both environmental and economic benefits. Currently, CWs have the potential to be restored using known and innovative land management practices, providing significant opportunities for carbon sequestration and CH_4_ offsetting. CH_4_ emissions from SFCWs have been found to be significantly higher than from SSFCWs, while CH_4_ emissions from VSSFCWs were significantly lower than from HSSFCWs. Several species-specific communities have been shown to facilitate the operation of energy-efficient and low-emission CWs. In addition, the selection of an appropriate substrate is also critical for CH_4_ mitigation. In CW-MFCs, the combination of biological and bio-electrochemical methods can effectively control CH_4_ emissions from CWs, although it is essential to maintain a stable balance between the systems CH_4_ production rate and power generation capacity. In addition, the O_2_ supply strategy, carbon source concentrate, and salinity control are key operational aspects that should be optimized to reduce the CH_4_ production potential of the system.

As research has progressed in this field, tremendous advances have been made in the control of CH_4_ emissions from CWs. However, there are still some aspects that require further investigation:

- When construction budgets and operating conditions allow, the use of integrated CWs is more effective for wastewater treatment, and optimization of the design of integrated systems for CH_4_ reduction will increase the development potential of CWs.
- The effect of different plant species on CH_4_ emissions in CW systems requires further research, with the rational application of plant litter as a substrate, such as biochar, may be beneficial to the overall sustainability and low-environmental impact of the CWs.
- Different anode materials can function as distinct types of electron acceptors, influencing the oxidation of CH_4_ in CW-MFC systems. Therefore, the mechanism of effect anode materials on CH_4_ production and emissions requires further investigation.
- Despite CWs being an essential ecosystem, most of the previously reported results were obtained from controlled mesoscale experiments, resulting in the need for future research to include field assessments conducted over long time spans.

## Author contributions

GY was responsible for data curation, formal analysis, and investigation. JC wrote the manuscript draft. GW, HC, and JH contributed to conceptualization, formal analysis, and visualization. YL, WW, FS, YM, QW, MW, TL, ZS, JS, and ZY provided feedback on the manuscript. All authors contributed to the article and approved the submitted version.

## Funding

This work was supported by the Hunan Provincial Natural Science Foundation of China (Nos. 2021JJ30728, 2021JJ40596, and 2019JJ50672), the Scientific Research Projects of Ecology and Environment Department of Hunan (No. HBKT-2021012), and the Water Conservancy Science and Technology Project of Hunan Province (No. XSKJ2022068-03).

## Conflict of interest

WW and FS were employed by Hunan Pilot Yanghu Reclaimed Water Co., Ltd. YM was employed by Hunan Rongantai Ecological Technology Co., Ltd. QW and MW were employed by CCCC-TDC Environmental Engineering Co., Ltd. TL and ZS were employed by China Railway Wuju Group the First Engineering Co., Ltd.

The remaining authors declare that the research was conducted in the absence of any commercial or financial relationships that could be construed as a potential conflict of interest.

## Publisher’s note

All claims expressed in this article are solely those of the authors and do not necessarily represent those of their affiliated organizations, or those of the publisher, the editors and the reviewers. Any product that may be evaluated in this article, or claim that may be made by its manufacturer, is not guaranteed or endorsed by the publisher.
